# Incidentally portal vein penetration during cannulation in endoscopic retrograde cholangiopancreatography: a case report

**DOI:** 10.1093/jscr/rjad298

**Published:** 2023-06-05

**Authors:** Pei-Yi Lin, Su-Hung Wang, Yu-Feng Tian, Ming-Jenn Chen, Ding-Ping Sun, Junping Shiau, Khaa-Hoo Ong

**Affiliations:** Division of Gastroenterology and General Surgery, Department of Surgery, Chi-Mei Medical Center, No.901, Zhonghua Rd., Yongkang Dist., Tainan City 710, Taiwan (R.O.C.); Division of Hepatogastroenterology, Department of Internal Medicine, Chi-Mei Medical Center, No.901, Zhonghua Rd., Yongkang Dist., Tainan City 710, Taiwan (R.O.C.); Division of Gastroenterology and General Surgery, Department of Surgery, Chi-Mei Medical Center, No.901, Zhonghua Rd., Yongkang Dist., Tainan City 710, Taiwan (R.O.C.); Division of Gastroenterology and General Surgery, Department of Surgery, Chi-Mei Medical Center, No.901, Zhonghua Rd., Yongkang Dist., Tainan City 710, Taiwan (R.O.C.); Division of Gastroenterology and General Surgery, Department of Surgery, Chi-Mei Medical Center, No.901, Zhonghua Rd., Yongkang Dist., Tainan City 710, Taiwan (R.O.C.); Department of Surgery, Kaohsiung Medical University Hospital, Kaohsiung Medical University, No.100, Ziyou 1st Rd., Sanmin Dist., Kaohsiung City 807, Taiwan (R.O.C.); Division of Gastroenterology and General Surgery, Department of Surgery, Chi-Mei Medical Center, No.901, Zhonghua Rd., Yongkang Dist., Tainan City 710, Taiwan (R.O.C.)

## Abstract

Portal vein cannulation is a very rare complication of endoscopic retrograde cholangiopancreatography (ERCP). In most reported cases, the event was managed safely with immediate catheter, guidewire withdrawn and procedure termination. Here, we report an unusual case of portobiliary fistula created during ERCP. To our knowledge, this is the first report of such case managed with immediate surgical biliary exposure.

## INTRODUCTION

Endoscopic retrograde cholangiopancreatography (ERCP) is now a frequently performed diagnostic and therapeutic procedure for pancreatobiliary disease, and it is important to understand the potential complications and risks of it. The most frequent complications of ERCP are pancreatitis, cholangitis, hemorrhage, and duodenal perforation. Portal vein cannulation (PVC) was very uncommon on the other hand, with an incidence between 1 in 6000 and 1 in 8000 cases [1, 2]. Although it can theoretically result in hemorrhage, sepsis, portal vein thrombosis and air emboli [3], most reported cases were safely managed with immediate withdraw of the catheter and guidewire [3–6]. Procedure termination without further intervention didn’t cause serious problems.

We experienced iatrogenic portobiliary fistula during ERCP, and managed differently from all other reports with immediate surgical biliary exposure under the intension to remove common bile duct (CBD) stones and also check for collateral damage as well. This unique experience demonstrated that the stent made perforation of CBD and portal vein may shrink after stent removal, and wouldn’t cause leakage under usual portal and bile duct pressure. This explains why immediate removal of the stent without ductal or vascular repairment is reasonable and wouldn’t cause further complication in most cases.

## CASE REPORT

Our patient was a 34-year-old man with a medical history of the Sturge–Weber syndrome, esophageal varices bleeding and mental retardation. He was admitted through emergency room due to CBD stones caused cholangitis.

**Figure 1:**
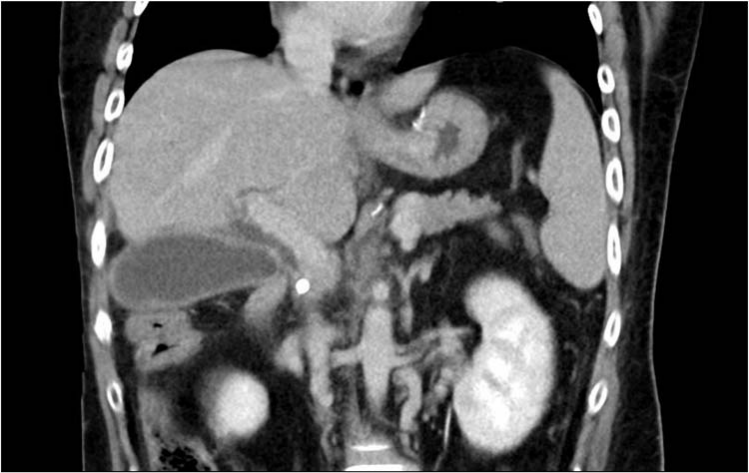
Abdominal CT revealed CBD stone resulting in upstream biliary dilatation and acute cholecystitis.

Initial laboratory findings revealed significant hyperbilirubinemia, liver enzyme elevation and mild PT prolongation. Abdominal computed tomography showed a CBD stone caused obstruction, resulting in upstream biliary dilatation and acute cholecystitis (Fig. 1). Besides, small caliber of inferior vena cava, splenic vein, and right iliac veins with thrombosis and prominent collaterals formation was also shown on the image.

**Figure 2:**
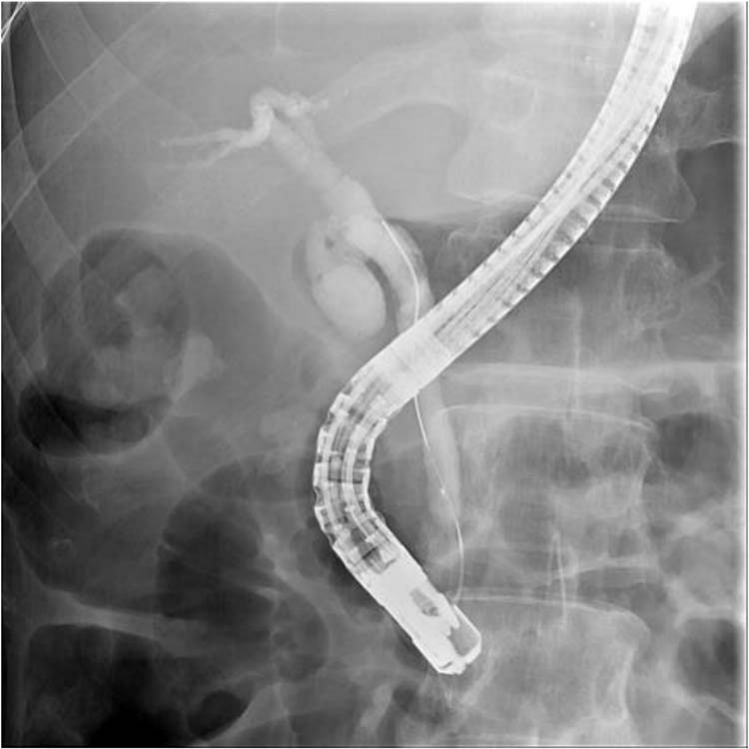
Successful wireguide CBD cannulation to pass through the opacified CBD stone following by cholangiography.

ERCP with a sphincterotomy was performed. After several attempts of wire-guided cannulation, successful CBD cannulation was achieved and confirmed by the cholangiography. Due to distal CBD stricture, we then used endoscopic balloon dilation repetitive of trying, we assumed deep cannulation to pass through the CBD stone was achieved because the guide wire advanced smoothly (Fig. 2).

**Figure 3:**
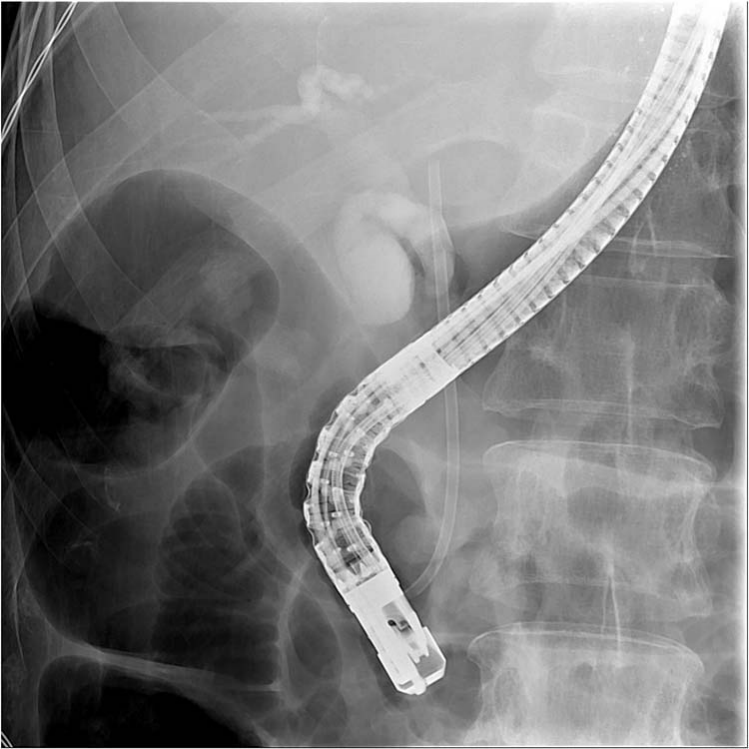
Fluoroscopy image showed the plastic stent penetrated through the CBD.

**Figure 4:**
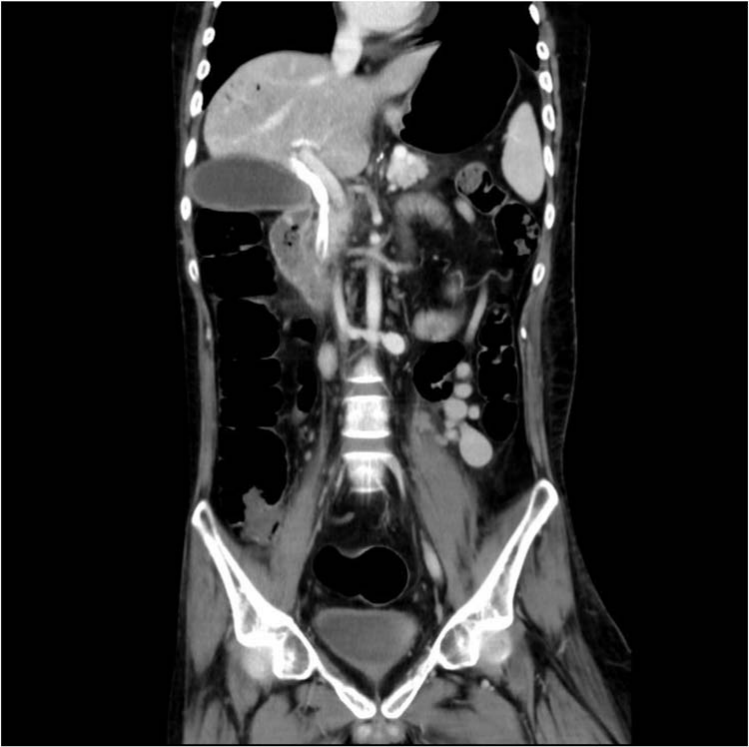
Just after ERCP, the abdominal CT showed retained contrast medium in bile duct.

**Figure 5:**
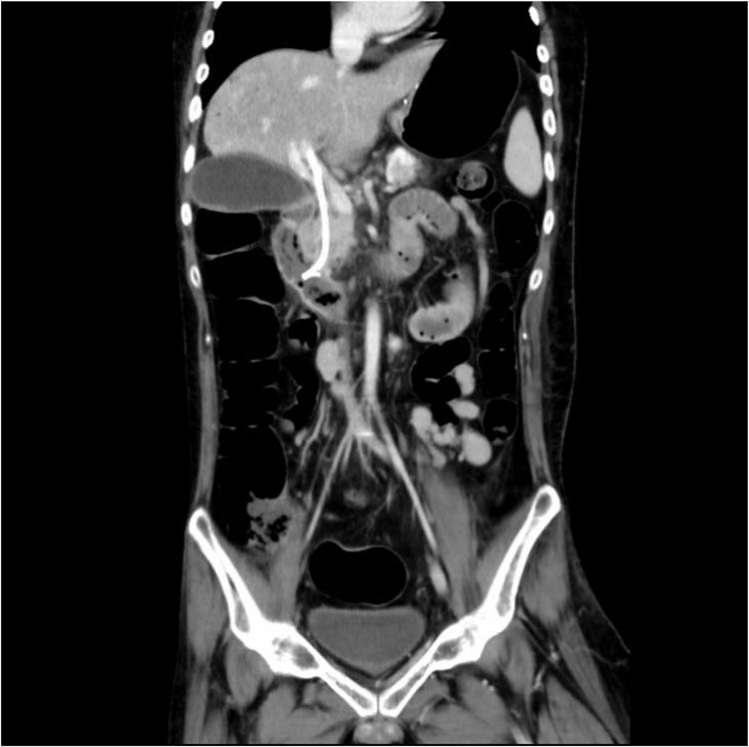
Malposition of the CBD internal stent with upper portion in main portal vein.

And then, a 7 French bostone scientific plastic stent was inserted over the guide wire for bile diversion. However, hemobilia was noted to be draining out from the stent right after. After reviewing the image, we realised that PVC was probably made (Fig. 3). After discussing with the general surgeon, we decided to arrange immediate surgical exploration to check for collateral damage and remove the CBD stones for cholangitis resolution. The stent was thus left inside for surgical guidance. Before the surgery, abdomen computed tomography (CT) was done and revealed retained contrast medium in the bile duct (Fig. 4) and malposition of the CBD internal stent with upper portion in the main portal vein (Fig. 5).

During the surgery, cholecystectomy was performed through the subcostal incision. Then the choledochoscope was inserted through the cystic duct stump to remove the stone in middle third of the CBD. Following the Kocher maneuver, we identified the internal stent penetrated through the CBD and into the portal vein at the intrapancreas level, with its tip at 1 cm above the gastroduodenal artery level. After we confirmed that no associated artery or organ injury was made, the malpositioned stent was removed by the gastroenterologist cooperatively through esophagogastroduodenoscopy. A 5 french nasogastric tube was then inserted through the cystic duct stump into distal CBD as a new stent. There was no vascular repair done and no hemorrhage or bile leakage was noticed intra-operatively under the surgeon’s surveillance.

After the operation, the patient overcame sepsis after 2-week treatment in the intensive care unit. Without bleeding episodes, he was discharged and remained uneventful for 9 months after the surgery.

## DISCUSSION

PVC is a very rare complication of ERCP, with an incidence between 1 in 6000 and 1 in 8000 cases [1, 2]. It can result from the laceration of a small portal vein or from a direct trauma to the papilla, extracorporeal shock wave lithotripsy caused pancreatic duct injury, and neo-angiogenesis or aberrant vessels development resulting from cancer or infection can also explain their occurrence [3, 4]. In this case, our patient has underlying disease of portosystemic collateral formation and ERCP was performed under acute cholangitis status. Furthermore, sphincterotomy and several guidewire cannulation attempts were made due to difficult cannulation resulted from CBD stricture. These may all facilitate PVC to happen.

Although portobiliary fistula carries a potential risk of hemorrhage, sepsis, portal vein thrombosis and air emboli [3], most reported cases were safely managed with immediate withdraw of the catheter and guidewire [3–6]. Procedure termination without further intervention didn’t cause serious problems. To our knowledge, there was only one reported case managed with successful biliary stenting through ERCP immediately after PVC [7]. And we are the only case managed differently from all others with surgical biliary exploration.

The decision was made under the intention to treat cholangitis with progressive sepsis from the origin. Redo-ERCP to remove the CBD stones is difficult and carries high risk of re-perforation according to the previous attempts. Surgical exploration made us able to remove the CBD stones and the inflamed gallbladder, and also check for, even repair the collateral damage if needed at the same time. We identified the stent penetrated through the CBD and into the portal vein intra-operatively. Fortunately, no artery or hollow organ was injured. After we removed the CBD stone by using choledochoscopy, the penetrated stent was removed by the gastroenterologist cooperatively through esophagogastroduodenoscopy. Another CBD stent was then inserted. Without ductal repair done, no hemorrhage or bile leakage was noticed intra-operatively. Our findings correlate with previously reported cases, demonstrating that the PVC can be safely managed with immediate catheter withdraw in most cases. The catheter induced perforation of portal vein and bile duct might shrink to seal spontaneously even without repair done, and wouldn’t cause leakage under usual portal and bile duct pressure. This explains why immediate removal of the stent without ductal or vascular repairment is reasonable and rarely causes further complication.

To avoid PVC, ERCP must be performed more carefully in patients with carcinoma or biliary tract inflammation. The occurrence can be safely managed with either immediate catheter withdraw, biliary stenting or surgical exploration under different circumstances. To our knowledge, this is the first report of such case managed with immediate surgical biliary exposure.

## DECLARATION

All authors certify that they have participated sufficiently in the work to take public responsibility for the content, including participation in the concept, design, analysis, writing or revision of the manuscript. Furthermore, each author certifies that this material or similar material has not been and will not be submitted to or published in any other publication.
